# Effectiveness of Positive Deviance Approach to Reduce Malnutrition among under Five Children: A Systematic Review and Meta-Analysis of Interventional Studies

**DOI:** 10.3390/nu15081961

**Published:** 2023-04-19

**Authors:** Nining Tyas Triatmaja, Trias Mahmudiono, Abdullah Al Mamun, Nurul Ashikin Abdullah

**Affiliations:** 1 Doctorate Degree Program in Public Health, Faculty of Public Health, Airlangga University, Surabaya 60115, Indonesia; nining.tyas.triatmaja-2022@fkm.unair.ac.id (N.T.T.); abdullah.al.mamun-2022@fkm.unair.ac.id (A.A.M.); 2 Department of Nutrition, Faculty of Public Health, Airlangga University, Surabaya 60115, Indonesia; 3 Institute Biological and Science, Faculty of Science, University of Malaya, Kuala Lumpur 50603, Malaysia; shikin84@um.edu.my

**Keywords:** malnutrition, positive deviance, stunting, under-five children, wasting

## Abstract

The high malnutrition rate in children under five makes this problem one of the public health problems. Various efforts have been made to reduce malnutrition in children under five, one of which is the implementation of community programs with a positive deviance approach which is considered an effective way because the solution to the problem comes from the local community. Thus, we conducted a systematic review and meta-analysis to determine the effect of interventions with a positive deviance approach to reducing under-five children’s malnutrition. Systematic searches were conducted using the following journal databases: Science Direct, Pubmed, Proquest, SAGE journal, Web of Science, and Scopus. The article was included if using an intervention design. Data analysis used Review Manager 5.4 software, random effect model, outcome mean of difference, and 95% confidence interval. There were no significant differences between the intervention and control groups on the length for age z-scores (LAZ), weight for age z-scores (WAZ), and weight for height z-scores (WHZ) indicators. There was an increase in LAZ, WAZ, and WHZ scores in the intervention group, with a greater z-score than in the control group. In conclusion, interventions with the positive deviance approach can be used as an alternative to improving the nutritional status of under-five children. However, further research is needed to determine the effective duration of interventions in improving the nutritional status of children.

## 1. Introduction

The high rate of stunting in under-five children worldwide, which was 33.7% in 2022, makes stunting one of the nutritional problems that receives critical attention [1]. Other nutritional problems, such as wasting, are still experienced by under-five children, namely around 47 million under-five children in 2020. Various efforts were made to reduce the prevalence of malnutrition, and it was proven that the stunting and wasting rate in under-five children decreased compared to previous years [2]. Malnutrition in under-five children causes a burden on the country’s economy due to decreased work productivity in adulthood, increased morbidity and mortality [3], and poor brain development [4]. Stunted children have a 5.48 times higher risk of mortality than non-stunted children due to infection and severe wasting increased the risk of death 11.63 times higher than that of under-five children with normal nutritional status [5]. Stunting in childhood impacts metabolic syndrome in adulthood [6] and tends to accumulate high visceral fat compared to subcutaneous fat and muscle mass [7]. Malnutrition in under-five children is caused by many factors, one of which is limited family resources. Limited family resources, such as poor family food diversity [8] and food insecurity, are associated with stunting [9]. Malnutrition in under-five children is also caused by inappropriate feeding practices such as non-exclusive breastfeeding, low dietary diversity, and low animal protein intake [10]. Children who did not consume animal protein had a risk of stunting 1436 higher than children who consumed animal protein [11]. Inappropriate children feeding is related with low maternal education [12].

Various efforts have been made to reduce the prevalence of malnutrition in children, both with sensitive and specific interventions. Comprehensive nutrition interventions incorporating nutrition education activities for maternal behavior change related to monitoring child growth and development and feeding for children have been shown to significantly improve children’s nutritional status [8]. Comprehensive intervention can be performed through a positive deviance approach. Positive deviance is an uncommon condition that indicates the behavior or activity of an individual or group in a population that [13] unconsciously becomes a protective factor against a particular problem [14]. Positive deviance behavior needs to be studied in a population because it can be a protective factor in health problems, such as malnutrition in under-five children. The behavior of the positive deviance group has unique characteristics that distinguish it from the non-deviance group. For example, mothers have high motivation and autonomy in feeding children and rarely provide food unsuitable for children [10]. Positive deviance families provide diverse and nutritious foods such as spinach, green vegetables, pumpkin, meat, fish, and shrimp that can be obtained in the river around the house [15]. Positive deviance behavior in various regions varies depending on the local potential that exists in the region.

The positive deviance approach includes positive deviance inquiry (PDI) to identify specific behavior of positive deviance family, mobilize communities to engage in planning and implementing educational programs (Hearth sessions), and behavior change through skill transferred from positive deviance family role modes [16]. It is the most effective way to fix public health problems because the solution to these problems is in the community [17] and utilizes available local resources so that they can be sustainable [18]. The positive deviance approach has been implemented in many countries [19,20,21,22], and there has been a systematic review publication to evaluate malnutrition reduction programs in children using a positive deviance approach [23]. The publication has not used a specific study design, and no meta-analytical studies related to positive deviance interventions have been conducted. Therefore, this systematic review and meta-analysis study aims to analyze the effectiveness of community programs using a positive deviance approach in reducing malnutrition problems in under-five children.

## 2. Materials and Methods

### 2.1. Data Sources, Search Strategies, and Serach Process

The study was based on Preferred Reporting Items for Systematic Reviews and Meta-analyses checklist guidelines (PRISMA) [24]. The article search was conducted on 26 August–4 September 2022 and was carried out by the first researcher. This study uses several journal databases: Science Direct, Pubmed, Proquest, SAGE journal, Web of Science, and Scopus. Our systematic review has registered in Prospero with No ID CRD42023413091. In addition, the study also used a manual search. This systematic review and meta-analysis study focused on intervention studies using two groups, namely the control and intervention groups. There is no limit on the publication date of this study. Search strategies on database journals are presented in Table 1. 

### 2.2. Inclusion and Exclusion Criteria

The general inclusion criteria set out in this study include (1) an original article, (2) it is in English, (3) interventional research, and (4) open access, while the exclusion criteria are set, namely (1) the article is in the form of a review, systematic review, or meta-analysis. The articles included in this systematic review and analysis study are stratified based on PICOS: participants/population, intervention and comparison, outcome, and study design. A record is entered if the participant/population is an under-five children. Based on the intervention, there are intervention groups (community programs with a positive deviance approach) and control. The record will be excluded if there is no control group. Based on outcomes, records will be included if the outcomes are length for age z-score (LAZ), weight for age z-score (WAZ), and weight for length or height z-score (WHZ).

### 2.3. Data Extraction

The first step in extracting data is to determine the same article (duplicate) based on the article title, and then the duplicate article is deleted. The next step is to evaluate the article by title and abstract. Titles and abstracts not eligible under inclusion and exclusion criteria are removed. Papers that met the inclusion and exclusion criteria are reviewed (full text).

### 2.4. Risk of Bias

Bias risk for randomized intervention research can be determined using the Cochrane risk-of-bias tool for randomized trials (RoB 2), Cochrane reviews. The risk of bias based on RoB 2 tools comes from (1) randomization process, (2) deviation derived from intervention, (3) bias due to missing outcome data, (4) bias in outcome measurement, and (5) selection bias of reported results. Based on these criteria, the risk of bias is categorized into 3, namely (1) low risk of bias, (2) some concerns, and (3) high risk of bias [25].

### 2.5. Statistical Analysis

Analysis of the data set searched using the software Review Manager 5.4, The Cochrane Collaboration, Copenhagen, Denmark. The analyzed data is continuous, so the outcome of the meta-analysis results is the mean of difference and 95% confidence interval [26]. The data results are said to be significant if the *p*-value < 0.05. The heterogeneity test also uses the same software and is indicated by *I*^2^ statistics. If the *I*^2^ number is >50%, the collected data are heterogeneous, so the random effect model will be used for data analysis. Conversely, if the *I*^2^ number ≤ 50%, the collected data are homogeneous, so a fixed effect model will be used [27]. The meta-analysis result data are presented in a forest plot, and publication bias is presented in a funnel plot.

### 2.6. Ethical Clearence

This study was exempted from IRB (Institutional Review Board) at the Faculty of Public Health, namely Komite Etik Penelitian Kesehatan (KEPK), Faculty of Public Health, Airlangga University, because all the data were obtained from open-access articles and databases.

## 3. Results

### 3.1. Database Search

**Figure 1:**
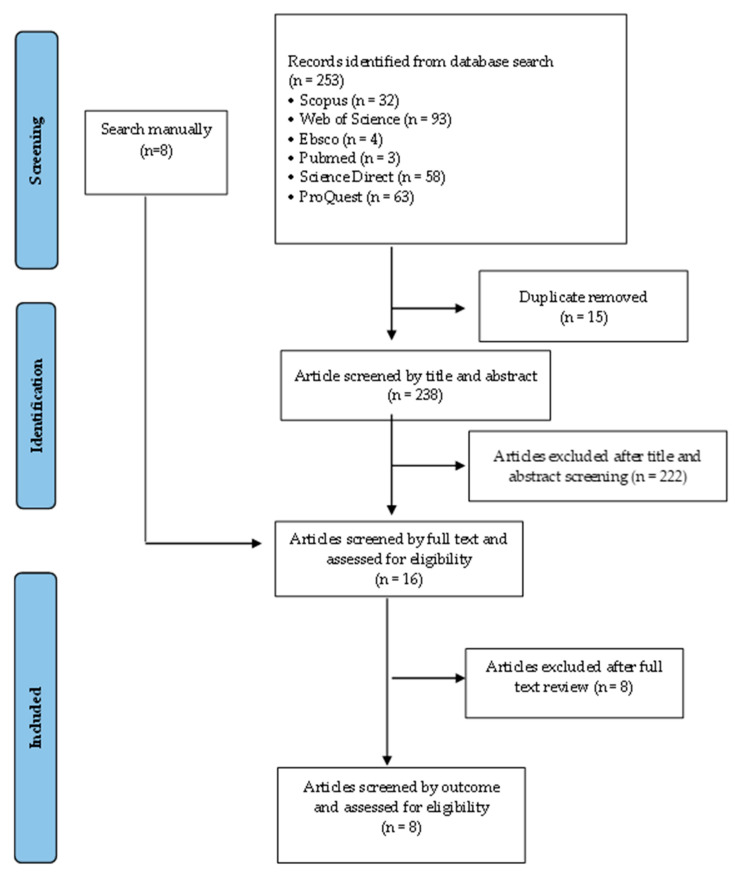
PRISMA flow diagram identification of articles included in systematic review and meta-analysis.

A total of 253 articles were obtained from searches on 6 databases using predetermined keywords, and 8 other articles were obtained from manual searches. A total of 15 articles were excluded due to duplication, and as many as 33 articles were recorded after screening titles and abstracts. A total of 25 articles were excluded because they did not show the outcome of LAZ, WAZ, and WLZ scores (*n* = 8). A total of eight articles were included in the systematic review, and only four were included in the meta-analysis. The articles cannot be included in the meta-analysis because they do not show mean and standard deviation data. Correspondence has been performed via email, but there has been no response. The identification of the search article is presented in the PRISMA flow diagram (Figure 1).

### 3.2. Study Description

The studies included in this meta-analysis amount to four articles [28,29,30,31]. The number of samples included in the analysis was 2467 under-five children. The description of the studies included in this meta-analysis is presented in Table 2. Kang et al. [28] conducted a study in Ethiopia involving 1125 respondents aged 6–24 months. The intervention provided was a community-based participatory nutrition promotion (CPNP) program adapting a Positive Deviance/Hearth approach for 2 weeks with a follow-up period of 12 months. The control group in the article is given a government program that is generally carried out in the region. The average age of the sample in the intervention and control groups was 8.7 months. The results of the intervention showed that children in the intervention group had a growth speed of body length [diff: 0.059 cm/month; 95% CI: 0.027, 0.092; *p* = 0.001] and body weight (diff: 0.031 kg/month; 95% CI: 0.019, 0.042; *p* < 0.001) that was faster than the control group. In addition, there was a decrease in the prevalence of stunting and underweight, which was 8.1% and 6.3%, respectively, at the end of the 12-month follow-up.

The second study included in this meta-analysis was conducted in Vietnam. Schroeder et al. conducted intervention research with a longitudinal, randomized controlled design. The intervention was carried out for 12 months, with the number of samples involving as many as 240 children aged 5–36 months. The average age of the samples in this study was 15.5 months. The study results from the article showed no significant differences in growth improvement between the control and intervention groups [29]. The third study included in the meta-analysis was conducted in Cambodia involving 330 children aged 6–23 months. The study used a randomized controlled trial (RCT) design that divided the sample into three groups, namely (1) the control group, (2) the traditional Positive Deviance/Hearth (PDH) program with in-person visits, and (3) the Positive Deviance/Hearth with Interactive Voice Calling (PDH-IVC) program group. However, the data used in this meta-analysis are from two groups: the control group and the traditional Positive Deviance/Hearth (PDH) program group. The result of the research from the article is that there was an improvement in WAZ values in the PDH and PDH-IVC groups during the 3-month follow-up, but these improvements persisted only in the PDH-IVC group [30]. Another study in Kenya involved 107 under-five children aged 6–59 months. The study results showed a decrease in mild and moderate underweights.

### 3.3. Risk of Bias

**Figure 2:**
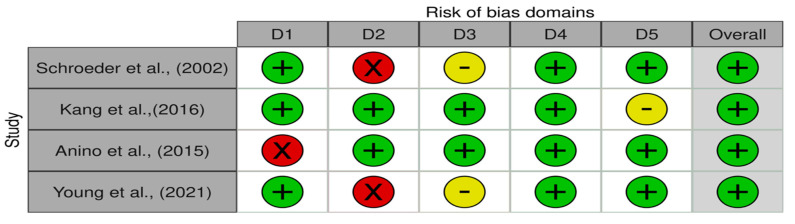
Assessment of the quality of articles included in the meta-analysis [28,29,30,31]. Domains: D1 = bias arising from the randomization process; D2 = bias due to deviations from the intended intervention; D3 = bias due to missing outcome data; D4 = bias in the measurement of the outcome; D5 = bias in the selection of the reported result.

**Figure 3:**
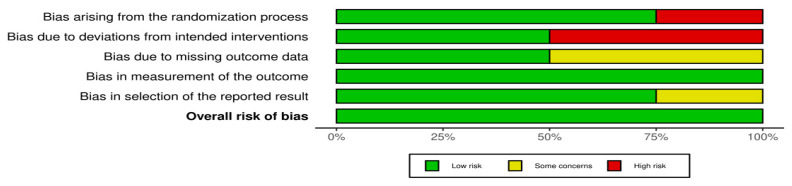
Assessment of the quality of articles (percentages) included in the meta-analysis [28,29,30,31].

All studies have a low risk of bias in terms of the measurement of the outcome. Domain randomization process shows that research [28,29,30] has a low risk of bias while other studies have a high risk of bias. High risk bias of domain randomization process in research [31] was caused by non-randomized trial in the research design. Research [25,28] shows low risk in the domain bias due to deviations from the intended intervention. High risk bias of domain intended intervention in research [29,30] was caused by participants and people of delivering intervention awareness during the trial. Overall, the research included in this meta-analysis has a low risk of bias. An assessment of the quality of the articles included in the meta-analysis is presented in Figure 2 and Figure 3.

### 3.4. Meta-Analysis Results

#### 3.4.1. Effect of Positive Deviance-Based Interventions on Length for Age Z-Score (LAZ)

Meta-analysis to determine the effect of positive deviance-based interventions on the length for age z-score (LAZ) was carried out in 3 research articles [28,29,30] with the number of samples in the intervention group as many as 735 and the control group as many as 839. Research by Kang et al. [28] showed a mean difference of 0.07 which means that the average sample in the intervention group experienced an increase in LAZ 0.07 points higher than the control group. However, the study of Schroeder et al. [29] showed that the average sample had a higher increase in LAZ in the control group than in the intervention group. The research of Young et al. [30] showed that the average sample in the intervention group had an increase in LAZ 0.92 points higher than the control group. 

**Figure 4:**
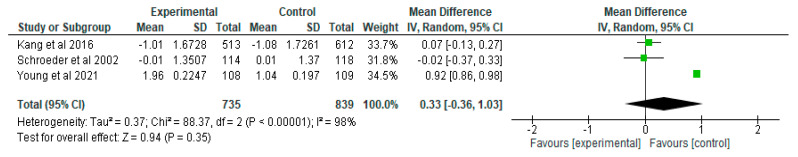
Results pooled data on the effect of positive deviance interventions on LAZ [28,29,30].

Meta-analysis results from three RCT studies showed that there was no significant difference in LAZ change between the positive deviance-based intervention group and the control group ((MD 0.33 z-score [95% CI −0.36, 1.03], random effects model, *p* = 0.35; 3 studies, *n* = 1574). The meta-analysis showed that the intervention group could improve the LAZ score by 0.33. There was heterogeneity in the three studies shown by I2 =98% (Figure 4).

#### 3.4.2. Effect of Positive Deviance-Based Interventions on Weight for Age Z-Score (WAZ)

Meta-analysis to determine the effect of positive deviance-based interventions on weight for age z-score (WAZ) was carried out in 4 research articles [28,29,30,31] with the number of samples in the intervention group as many as 776 and the control group as many as 905. Research by Kang et al. [28] showed a mean difference of 0.27 which means that the average sample in the intervention group experienced an increase in WAZ 0.27 points higher than the control group. The same was true of the 2015 study of Anino et al. [31] which showed a 0.81-point increase in WAZ in the intervention group than in the control group. However, the study of Schroeder et al. [29] showed that the average sample had a higher WAZ increase in the control group than the intervention group. The research of Young et al. [30] showed that the average sample in the intervention group had an increase in WAZ 0.08 points higher than the control group.

**Figure 5:**
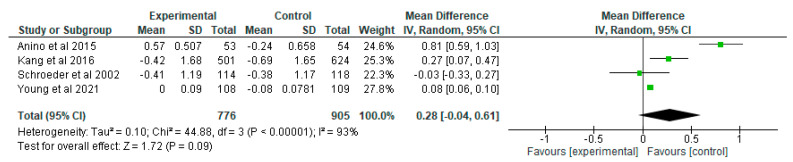
Results pooled data on the effect of positive deviance interventions on WAZ [28,29,30,31].

Meta-analysis results from four intervention studies showed that there was no significant difference in WAZ changes between the positive deviance-based intervention group and the control group (MD 0.28 z-score [95% CI −0.04, 0.61], random effects model, *p* = 0.09; 4 studies, *n* = 1681). The meta-analysis showed that the intervention group could improve the WAZ score by as much as 0.28. There was heterogeneity in the three studies shown by *I*^2^ = 93% (Figure 5).

#### 3.4.3. Effect of Positive Deviance-Based Interventions on Weight for Height Z-Score (WHZ)

**Figure 6:**
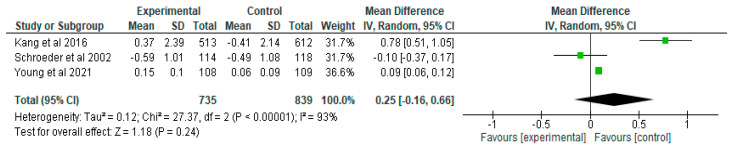
Results pooled data on the effect of positive deviance interventions on WHZ [28,29,30].

Meta-analysis to determine the effect of positive deviance-based interventions on weight for height z-score (WHZ) was carried out in 3 research articles [28,29,30] with the number of samples in the intervention group as many as 735 and the control group as many as 839. Similar to the LAZ and WAZ indicators, the pooled data in this meta-analysis showed no significant difference in WHZ change between the positive deviance-based intervention group and the control group (MD 0.25 z-score [95% CI −0.16, 0.66], random effects model, *p* = 0.24; 3 studies, *n* = 1574). There is heterogeneity in the three studies on the WHZ indicator (*I*^2^ = 93%) (Figure 6).

### 3.5. Risk of Publication Bias

**Figure 7:**
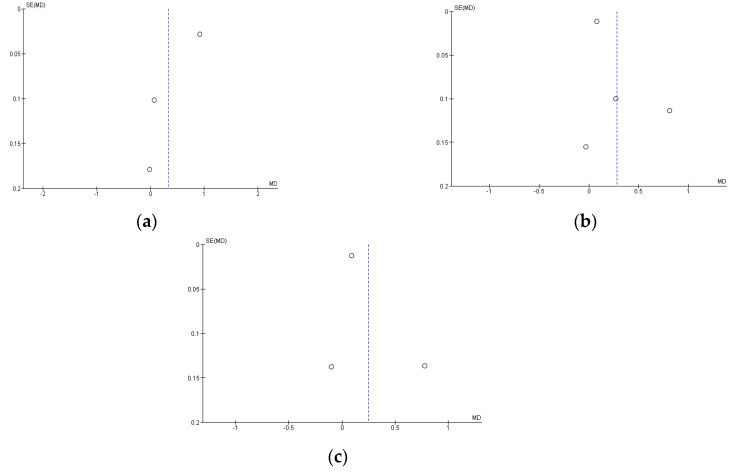
Risk assessment of publication bias using plot funnels with LAZ (**a**), WAZ (**b**), and WHZ (**c**) indicators.

The funnels plot of the LAZ, WAZ, and WLZ indicators are presented in Figure 7, all three of which show asymmetrical funnel plots. High heterogeneity can lead to asymmetric plot funnels [35]. There is a statistical test to assess the asymmetry of the funnel plot, namely Egger’s [36] test. Still, the test cannot be used if only <10 articles are included in the meta-analysis [35].

## 4. Discussion

The high malnutrition rate in under-five children makes the problem one of the priorities global governments address. One of the efforts that can be made is imitating good behavior carried out by groups of under-five children populations that do not experience malnutrition. This approach is a positive deviance approach that has been carried out in many countries. Several studies have developed new intervention methods based on positive deviance approaches such as mobile phone-based Positive Deviance/Hearth program [30] and home visiting-based positive deviance program [34].

Community programs that use a positive deviance approach are proven to improve the nutritional status of under-five children. The prevalence of underweight in Cambodia’s positive deviance-based intervention group decreased by 12.8% compared to the control group [30]. A positive deviance-based community program conducted in South Africa also showed positive results for under-five children weight gain [33]. Improvement of the nutritional status of under-five children through a positive deviance approach occurs due to an increase in food intake given in the intervention group [21,37] and an increase in maternal and caregiver awareness in feeding [32]. Interventions using positive deviance also can improve risk factors of malnutrition such as hygiene practices among caregivers [20]. Positive deviance approaches are generally affordable, acceptable, and sustainable because they are practiced by at-risk people and there is no conflict with local culture [38]. This intervention requires high community awareness to participate in this intervention because it needs social mobilization, information gathering, and behavioral change [39]. The other challenges of this approach are identification of specific practice or positive deviance behavior through positive deviance inquiry (PDI). PDI needs more contact with positive deviance family to obtain quality data [16]. The meta-analysis aims to analyze the effectiveness of interventions with a positive deviance approach to the incidence of malnutrition in under-five children. This meta-analysis showed no significant difference between the group given positive deviance-based interventions and the control group regarding all nutritional status indicators tested, namely LAZ, WAZ, and WHZ. This is in accordance with research conducted in Malawi which shows that positive deviance-based interventions have no significant effect on LAZ. Stunting improvement takes a long time, and mothers and caregivers must consistently serve food according to what has been taught during the intervention [32]. The average duration of intervention carried out by researchers in this meta-analysis was 12 days, with a follow-up period of 6–12 months. Positive Deviance or Hearth Session behavioral interventions are carried out for 10–12 days and followed up for 2 weeks to ensure that the behavior is carried out for 21 days to make a new habit [40]. Future research needs to determine the duration of effective interventions to improve maternal behavior and the nutritional status of children.

This meta-analysis showed no significant differences in WAZ and WLZ indicators between the intervention and control groups. The effect of positive deviance interventions is significant on under-five children weight gain likely to be age-related to under-five children when engaging in intervention programs. Studies conducted in Bangladesh show that under-five children of younger ages show significant WAZ improvements than under-five children of older ages. This is because caregivers pay more attention when the child is younger (<1 year old). In addition, the food served during the intervention session is likely more appropriate for young children [19].

This meta-analysis has several strengths: it only involves interventional research and implementing a comprehensive article search strategy. In addition to its strengths, this meta-analysis has the disadvantage of a high degree of heterogeneity in the articles included in the meta-analysis. The high heterogeneity in this meta-analysis can come from the sample selection criteria, the difference in sample age, the intervention duration, and the follow-up duration. This systematic review and meta-analysis were conducted based on PRISMA guidelines with inclusion and exclusion criteria in articles included in the systematic review and meta-analysis. This aimed to minimize the risk of bias and increase trustworthiness of the chosen methods. Interventional research using the positive deviance approach is still limited, which can be a weakness in this meta-analysis. The data available in the article are limited. Researchers have attempted to contact the article’s author to obtain comprehensive data, but there has been no response.

## 5. Conclusions

The results of this meta-analysis show that interventions with a positive deviance approach can increase the value of LAZ, WAZ, and WHZ, even though there are no significant differences compared to the control group. Interventions using a positive deviance approach can be used as an alternative in treating malnutrition in under-five children. More research is needed to determine the duration of effective interventions to improve the nutritional status of under-five children.

## Figures and Tables

**Table 1:** Search strategy in selected database.

Database	Search Strategy	Number of Articles
Scopus	TITLE-ABS-KEY (“positive deviance intervention” OR “hearth” AND (“under five children stunting” OR “wasting” OR “underweight” OR “children malnutrition” OR “nutritional status” OR “child growth”)) AND (LIMIT-TO (LANGUAGE, “English”))	32
Web of Science	“positive deviance intervention” OR “hearth” AND (“under five children stunting” OR “wasting” OR “underweight” OR “children malnutrition” OR “nutritional status”	93
Proquest	“positive deviance intervention” OR “hearth intervention” AND “under five children stunting wasting underweight” OR “under five child malnutrition” OR “under five child nutritional status” NOT “literature review” NOT “systematic review” NOT “meta-analysis”	63
EBSCO	“positive deviance intervention” OR “hearth” AND (“under five children stunting” OR “wasting” OR “underweight” OR “child malnutrition” OR “nutritional status”)	4
Pubmed	“positive deviance intervention” OR “hearth” AND (“under five children stunting” OR “wasting” OR “underweight” OR “child malnutrition” OR “nutritional status”)	3
Science direct	“positive deviance” OR “hearth” AND “experimental trial” AND “under five children stunting” OR “under five children wasting” OR “under five children underweight” OR “under five child malnutrition” OR “under five child nutritional status”	58

**Table 2:** Summary of findings from the included studies.

Study	Study Design	Country	Sample (*n*)	Age	Intervention	Control	Duration	Outcome
Kang et al. (2016) [28]	community-based cluster randomized trial	Ethiopia	1790	6–24 month	a community-based participatory nutrition promotion (CPNP) program adapting a Positive Deviance/Hearth approach	community-based nutrition programs in poor rural districts	12 days intervention and 12 months follow-up	Mean of LAZ, WAZ, and LWZ
Schroeder et al. (2002) [29]	Longitudinal, randomized controlled design	Vietnam	232	5–36 month	Positive deviance program	No Program	12 days intervention and 12 months follow-up	Mean of LAZ, WAZ, and LWZ
Young et al. (2021) [30]	Longitudinal cluster randomized controlled trial	Kamboja	330	6–23 month	(1) traditional Positive Deviance/Hearth (PDH) program with in-person visits, (2) Positive Deviance/Hearth with Interactive Voice Calling (PDH-IVC) program	the standard government Basic Health and Nutrition Package	10 days intervention and 12 months follow-up	Mean of LAZ, WAZ, and LWZ
Anino et al. (2015) [31]	Quasy Experimental	Kenya	107	6–59 month	Positive deviance program	No Program	12 days intervention and 6 months follow-up	Mean of LAZ, WAZ, and LWZ
Seetha et al. (2018) [32]	Randomized controlled trial	Malawi	179	0–23 month	Positive deviance/Hearth model	No program	21 days	Difference of mean of LAZ, WAZ, and LWZ
Le Roux et al. (2011) [33]	Randomized controlled trial	Afrika Selatan	635	6–12 month	Positive deviance/Hearth model	No program	12 days intervention and 12 months follow-up	Mean of WAZ
Roche et al. (2016) [21]	Quasy experimental	Ecuador	264	0–23 month	Positive deviance/Hearth model	No program	12 days intervention and 6 months follow-up	Mean percentage of the recommended intake, Change of WAZ
Le Roux et al. (2010) [34]	Randomized controlled trial	South Africa	788	0–5 years old	Mentor mother with positive deviance	community-based nutrition programs from government	12 days intervention and 6 months follow-up	Weight rehabilitation

## Data Availability

Data supporting reported results can be directed to the corresponding authors.
